# Redetermination and new description of the crystal structure of vanthoffite, Na_6_Mg(SO_4_)_4_

**DOI:** 10.1107/S2056989020005873

**Published:** 2020-05-01

**Authors:** Tonči Balić-Žunić, Martha G. Pamato, Fabrizio Nestola

**Affiliations:** a University of Copenhagen, Department of Geosciences, Denmark; b Department of Geosciences, University of Padova, Italy

**Keywords:** crystal structure, vanthoffite, Na-Mg sulfate, atomic coordinations, redetermination

## Abstract

The crystal structure of vanthoffite {hexa­sodium magnesium tetra­kis[sulfate­(VI)]}, Na_6_Mg(SO_4_)_4_, was solved in the year 1964 on a synthetic sample [Fischer & Hellner (1964 ▸). *Acta Cryst.* **17**, 1613]. Here we report a redetermination of its crystal structure on a mineral sample with improved precision. It was refined in the space group *P*2_1_/*c* from a crystal originating from Surtsey, Iceland. The unique Mg (site symmetry ) and the two S atoms are in usual, only slightly distorted octa­hedral and tetra­hedral coordinations, respectively. The three independent Na atoms are in a distorted octa­hedral coordination (1×) and distorted 7-coordinations inter­mediate between a ‘split octa­hedron’ and a penta­gonal bipyramid (2×). [MgO_6_] coordination polyhedra inter­change with one half of the sulfate tetra­hedra in <011> chains forming a (100) meshed layer, with dimers formed by edge-sharing [NaO_7_] polyhedra filling the inter­chain spaces. The other [NaO_7_] polyhedra are organized in a parallel layer formed by [010] and [001] chains united through edge sharing and bonds to the remaining half of sulfate groups and to [NaO_6_] octa­hedra. The two types of layers inter­connect through tight bonding, which explains the lack of morphological characteristics typical of layered structures.

## Abstract 2

The crystal structure of vanthoffite, Na_6_Mg(SO_4_)_4_, was redetermined and refined with anisotropic displacement parameters for all atoms. Here, for the first time, we give its detailed description.

## Chemical context   

Vanthoffite is an evaporitic mineral that occurs worldwide in various salt, potash and sulfate marine deposits. It is also reported from the fumaroles of Kamchatka (Pekov *et al.*, 2015 ▸) and Iceland (Balić-Žunić *et al.*, 2016 ▸). Fischer & Hellner (1964 ▸) solved its crystal structure giving the crystal lattice parameters, space group and atomic coordinates with isotropic atomic displacement parameters. To the best of our knowledge the only other crystal structure determination and refinement of an isostructural compound is for Na_6_Mn(SO_4_)_4_ (Sharma *et al.*, 2017 ▸).

Here we report a redetermination and refinement of the crystal structure of vanthoffite, complete with anisotropic displacement parameters and provide a more detailed description. The precision of the present results is significantly better compared to the previous data of Fischer & Hellner (1964 ▸) because of the capabilities of modern X-ray diffraction equipment based on a hybrid photon-counting detector. The obtained *R* factor for the observed reflection data is 3.2% compared to 6.4% for the previous refinement, and the standard deviations of the atomic coordinates and displacement parameters are three to five times smaller. Consequently, the standard deviations of bond lengths and angles are generally ten or more times smaller than previously reported. Comparing our results with those of Fischer & Hellner (1964 ▸), we conclude that no differences of substantial character can be observed, and this pays a special credit to the latter work done with significantly more effort than needed for the present one. The improvement in precision that we obtained, however, allows us to evaluate important structural details that were until now lacking for vanthoffite.

## Structural commentary   

In this work, we use three distortion parameters for the description of deviations of atomic coordinations from an ideal geometrical arrangement, *viz.* asphericity, eccentricity and volume distortion, as defined by Balić-Žunić & Makovicky (1996 ▸), and Makovicky & Balić-Žunić (1998 ▸). Numerical values of these parameters are collated in Table 1 ▸. They are useful because they clearly define the type and reason for distortion (in the case of a Jahn–Teller effect or the presence of lone electron pairs), and at the same time define the closest type of the coordination polyhedron.

### Coordination polyhedra of Mg and S atoms   

**Figure 1:**
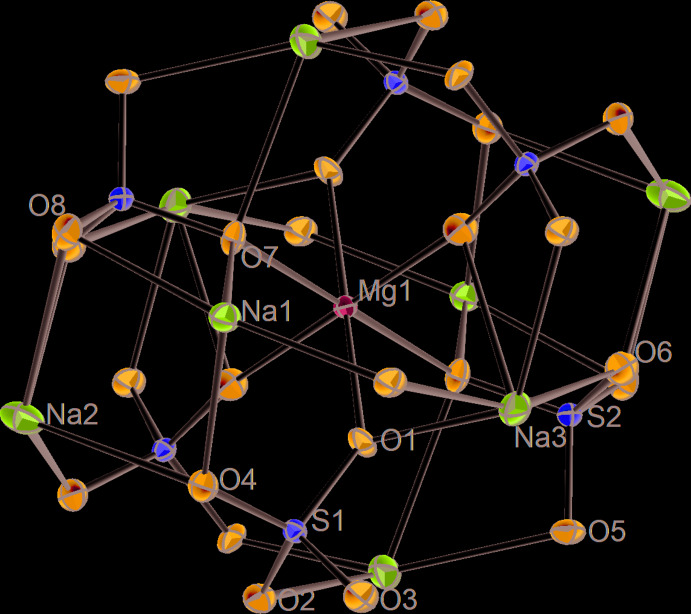
The atomic grouping around the [MgO_6_] coordination polyhedron. Anisotropic displacement ellipsoids are drawn at the 50% probability level.

The unique Mg atom is located on a symmetry centre and is octa­hedrally coordinated by O atoms, whereas the two independent S atoms form tetra­hedral sulfate groups with oxygen atoms (Fig. 1 ▸). The coordinations of Mg and S show very small distortions from the ideal octa­hedral and tetra­hedral arrangements, respectively. As can be seen from Table 1 ▸, both S coordination polyhedra have very similar parameters. They are slightly eccentric; the longest bonds are to the O atoms that they share with Mg. This is plausible, because Mg has a larger electronegativity and a higher charge than Na. The Mg coordination polyhedron is even less distorted than those of S. The eccentricity is zero, in accordance with the site being on a symmetry centre and the other two distortion parameters are very low. The anisotropy of the displacement parameters of oxygen atoms bonded to S and Mg, with the overall oblate character of their ellipsoids and the longest diameters approximately perpendicular to the bonding directions, suggests, together with a low anisotropy of S and Mg displacement parameters, a rotational displacement of the coord­ination polyhedra around their centres (Fig. 1 ▸). Each of the vertices of an [MgO_6_] octa­hedron is shared with one sulfate tetra­hedron, in an arrangement known as a pinwheel structure (Moore, 1973 ▸).

### Coordination polyhedra of Na atoms   

**Figure 2:**
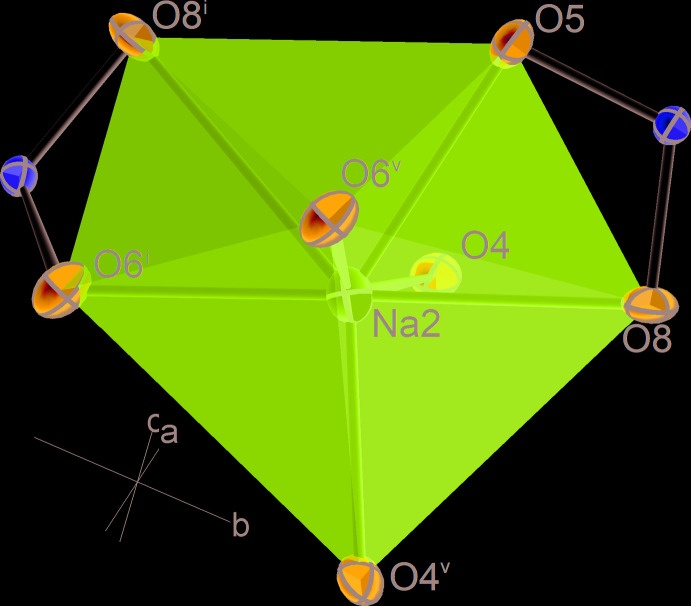
Atomic coordination of the Na2 atom. Displacement ellipsoids are as in Fig. 1 ▸. [Symmetry codes: (i) 1 − *x*, −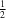 + *y*, 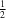 − *z*; (v) *x*, 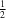 − *y*, −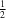 + *z*].

**Figure 3:**
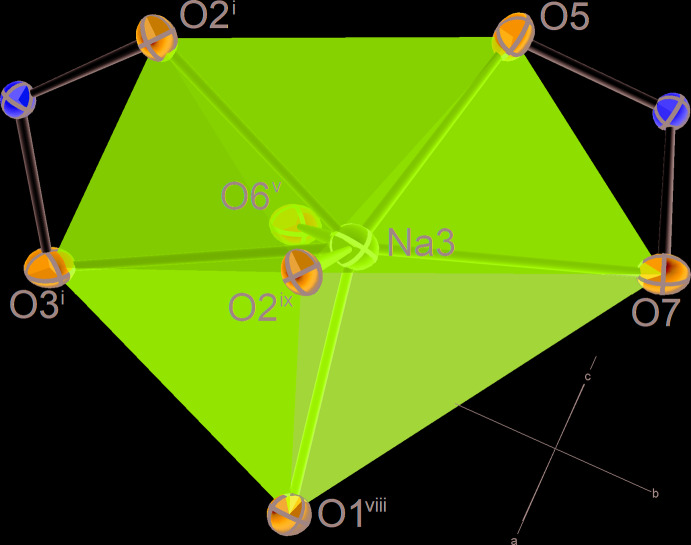
Atomic coordination of the Na3 atom. Displacement ellipsoids are as in Fig. 1 ▸. [Symmetry codes: (i) 1 − *x*, −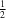 + *y*, 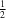 − *z*; (v) *x*, 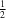 − *y*, −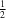 + *z*; (viii) 1 + *x*, *y*, *z*; (ix) 1 + *x*, 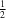 − *y*, 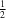 + *z*].

The coordination environment for Na atoms is distorted octa­hedral in the case of Na1 and split-octa­hedral with a coordination number (CN) of 7 for the other two independent Na sites (Figs. 2 ▸ and 3 ▸). We consider only the O atoms closer than 3 Å to be bonded to Na. There are further O atoms in the neighbourhood of Na, listed by Fischer & Hellner (1964 ▸), but we note that the distance gap to these additional O atoms is significant and their bonding contribution negligible according to bond-valence calculations. The volume distortions of the coordination polyhedra around Na2 and Na3 lie between those of an ideal penta­gonal bipyramid (0) and an ideal ‘split octa­hedron’ (0.1333). The latter type of coordination was described in detail by Edenharter (1976 ▸) and Makovicky & Balić-Žunić (1998 ▸). The coordination polyhedron of Na2 (Fig. 2 ▸) can either be described as a penta­gonal bipyramid with O4 and O6 as polar vertices, or as a split octa­hedron with O5 and O8 as a split vertex. Likewise, the coordination polyhedron of Na3 (Fig. 3 ▸) can either be described as a distorted penta­gonal bipyramid with O2 and O6 as polar vertices, or as a split octa­hedron with the second O2 and O5 as a split vertex. It can, furthermore, be seen from Figs. 2 ▸ and 3 ▸ that coordination polyhedra of Na2 and Na3 each share two edges with sulfate tetra­hedra. Edges O5–O8 and O6–O8 of [Na2O_7_] are shared with two [S2O_4_] tetra­hedra, whereas edges O2–O3 and O5–O7 of [Na3O_7_] are shared with a S1 and a S2 coordination tetra­hedron, respectively.

### Description of the crystal structure as an arrangement of coordination polyhedra of cations   

**Figure 4:**
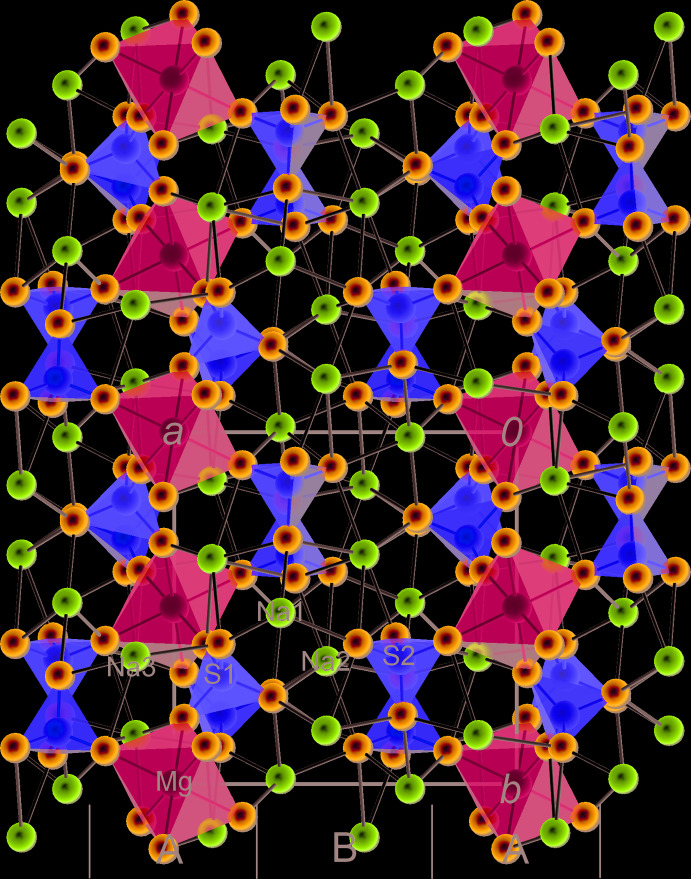
Projection of the crystal structure of vanthoffite along [001] with indication of the *A* and *B* structural layers.

**Figure 6:**
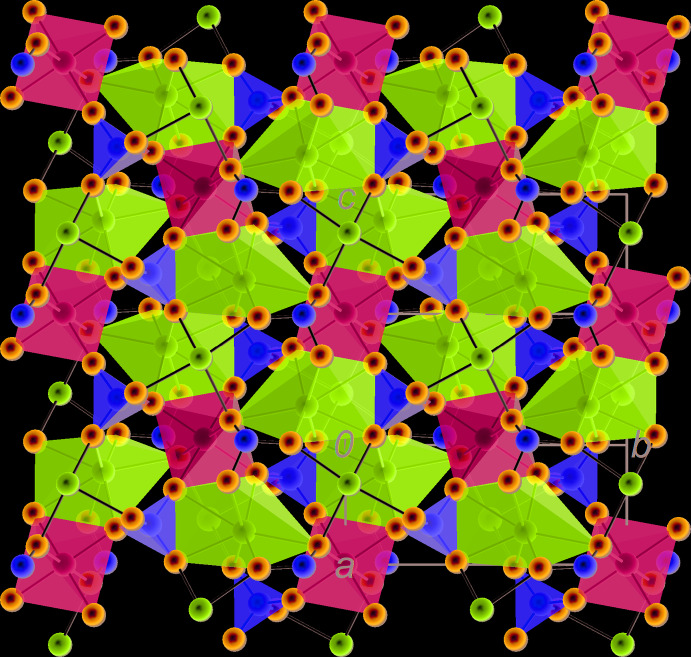
Layer *A* formed by the coordination polyhedra of Mg, S1 and Na3, projected on (100), with the *c* axis vertical. Attachment of S2 and Na1, both visualized as spheres, is shown.

The crystal structure of vanthoffite can be described as an inter­change of two types of layers parallel to {100}, here labelled *A* and *B* (Figs. 4 ▸ and 5 ▸). Layer *A* is centred on the (0, *y*, *z*) plane and built of coordination polyhedra of Mg, S1 and Na3 (Fig. 6 ▸). [MgO_6_] octa­hedra share four vertices with four [S1O_4_] tetra­hedra. They form inter­secting chains running along the <011> directions. Sharma *et al.* (2017 ▸) described the crystal structure of the vanthoffite type as having an infinite two-dimensional framework of Mg coordination polyhedra and sulphate groups in the *bc* plane (which we confirm), but describe this framework as being composed of inter­connected chains parallel to [010], which is an obvious mistake, as can be seen from Fig. 6 ▸. In layer *A*, [Na3O_7_] coordination polyhedra are arranged in pairs that share a common edge (O2–O2′). If we consider the coordination polyhedra of Mg and Na3 alone, they form chains parallel to [001] in which the Mg and Na coordination polyhedra also share edges (O1–O3). The chains inter­connect through common O7 vertices, belonging to both the Mg and Na3 coordination polyhedra. The [Na3O_7_] polyhedron also shares its O2–O3 edge with an [S1O_4_] tetra­hedron as mentioned above, plus an O1 vertex with another [S1O_4_] tetra­hedron.

**Figure 7:**
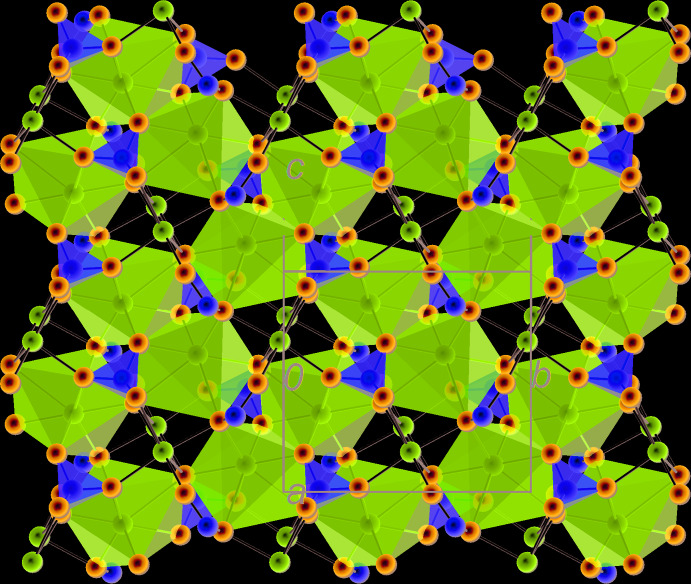
Layer *B* formed by the coordination polyhedra of Na1, Na2 and S2, projected on (100), with the *c* axis vertical. To enhance clarity, the coordination polyhedra of Na1 are not filled; attachment of S1 visualized as a sphere is shown.

Layer *B* is centred on the (1/2, y, z) plane and built of coordination polyhedra of Na1, Na2 and S2 (Fig. 7 ▸). [Na2O_7_] coordination polyhedra form chains around the *c* symmetry planes, by sharing O4 vertices. These chains run along [001]. They also form chains along [010] and around 2_1_ axes by sharing O8 vertices. The two types of chains inter­connect by sharing O6–O6′ edges, in the middle of which are situated symmetry centres. As mentioned above, [S2O_4_] tetra­hedra, located in this layer, share two edges (O5–O8 and O6–O8) with [Na2O_7_] polyhedra. Na1 atoms lie in distorted octa­hedral holes formed between neighbouring *A* and *B* layers. The corresponding [Na1O_6_] octa­hedra share O4–O8 and O5–O8 edges with [Na2O_7_] and O2–O5 edges with [Na3O_7_]. There are further inter­connections between the two types of layers (O5–O6 edges shared by Na3 and Na2, O5–O7 edges shared by Na3 and S2, plus several shared vertices).

Fischer & Hellner (1964 ▸) described the crystal structure of vanthoffite as a distorted hexa­gonal close packing of sulfate groups, with Mg in ¼ of the octa­hedral holes. The authors did not specify the orientation of the close-packed sulfate layers. There are indeed approximately eutactic layers of sulfate groups parallel to the (001) plane. Their composition and stacking, however, deviate considerably from an ideal eutaxy. Moreover, considering the full framework of coordination polyhedra and chemical bonds, the structure is best described as layered parallel to (100) as in this work and in Hawthorne *et al.* (2000 ▸). Most of the previous authors essentially ignored the function of the [NaO_*x*_] coordination polyhedra in building the crystal structure, and just mentioned the placement of Na in the holes of the framework of the Mg and S coordination polyhedra. Only Fischer (1973 ▸) discussed the three Na coordination types in this structure in a conference abstract. Since Na is the dominating cation in vanthoffite, the structure-building role of the [NaO_*x*_] coordination polyhedra also needs to be considered, as we have tried to do in this article.

Vanthoffite is characterized by having six times as many Na atoms as Mg ones. The availability of Na coordination polyhedra in close contact, defining a three-dimensional framework, makes it a potential Na^+^ ionic conductor. Sharma *et al.* (2017 ▸) found a high Na^+^ conductivity only in the material obtained after the transition of vanthoffite-type Na_6_Mn(SO_4_)_4_ to a high-temperature phase. We hope that the present detailed description can help in understanding why Na^+^ conductivity is observed in the high-temperature form only (once its structure is known), but not in the vanthoffite structure itself.

## Synthesis and crystallization   

The crystal used for the crystal structure analysis originates from a sample from Surtsey, collected in 1971 by Dr Svein Peter Jakobsson from the Icelandic Institute of Natural History, four years after the end of eruption that formed this volcanic island. The sample number in the mineral collection of the Institute is IN7484.

## Refinement   

Crystal data, data collection and structure refinement details are summarized in Table 2 ▸. Atomic sites were labelled to correspond to the original description of the crystal structure (Fischer & Hellner, 1964 ▸). A chemical analysis of the analysed crystal was not performed because of its very small size. As is typical for minerals from volcanic fumaroles, the mineral is fine grained and intimately mixed with several other phases, which makes an accurate chemical analysis extremely difficult, even on a larger sample. The correspondence of the current crystal-structure parameters to those of the synthetic compound and the results of structural refinement indicate that the chemical composition is indeed very close to ideal without apparent influence from chemical impurities.

## Supplementary Material

Crystal structure: contains datablock(s) I. DOI: 10.1107/S2056989020005873/wm5553sup1.cif

Structure factors: contains datablock(s) I. DOI: 10.1107/S2056989020005873/wm5553Isup2.hkl

Supporting information file. DOI: 10.1107/S2056989020005873/wm5553Isup3.cml

CCDC reference: 1999656

Additional supporting information:  crystallographic information; 3D view; checkCIF report

## Figures and Tables

**Figure 5:**
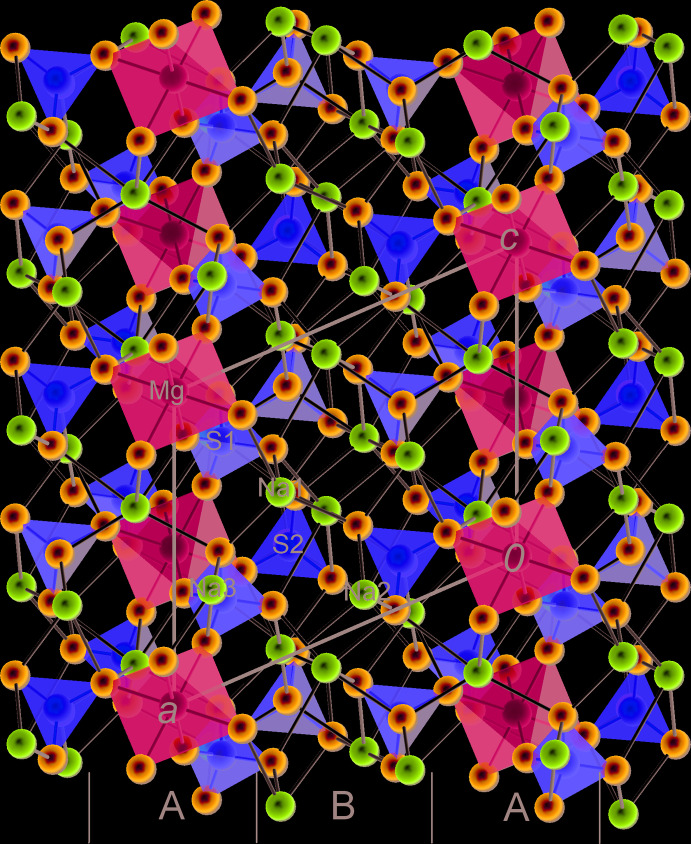
Projection of the crystal structure of vanthoffite along [010] with indication of the *A* and *B* structural layers.

**Table 1:** The parameters of the coordination polyhedra calculated with the program *IVTON* (Balić-Žunić & Vicković, 1996 ▸)

Cation	CN	<d> (Å)	bvs	Vp (Å^3^)	vd	asp	ecc
S1	4	1.474	6.002	1.643 (4)	0.0003	0	0.0115
S2	4	1.473	6.027	1.637 (4)	0.0006	0	0.0124
Mg	6	2.083	2.094	11.99 (2)	0.0046	0.0060	0
Na1	6	2.408	1.177	17.18 (2)	0.0716	0.0119	0.0392
Na2	7	2.561	1.074	24.64 (4)	0.0674	0.0586	0.0480
Na3	7	2.524	1.071	22.76 (3)	0.0957	0.0602	0.1492

Notes: CN = coordination number; <*d*> = average bond length; bvs = bond valence sum, calculated using the exponential function of Brown & Altermatt (1985 ▸) with the parameters of Brese & O’Keeffe (1991 ▸); *V*p = polyhedral volume; vd = volume distortion; asp = asphericity; ecc = eccentricity.

**Table 2:** Experimental details

Crystal data	
Chemical formula	Na_6_Mg(SO_4_)_4_
*M*_r_	546.5
Crystal system, space group	Monoclinic, *P*2_1_/*c*
Temperature (K)	296
*a*, *b*, *c* (Å)	9.7761 (6), 9.1998 (4), 8.2040 (5)
β (°)	113.518 (7)
*V* (Å^3^)	676.56 (7)
*Z*	2
Radiation type	Mo *K*α
μ (mm^−1^)	1.04
Crystal size (mm)	0.03 × 0.02 × 0.004

Data collection	
Diffractometer	SuperNova Rigaku Oxford Diffraction diffractometer with Pilatus200K detector
Absorption correction	Multi-scan (*CrysAlis PRO*; Rigaku OD, 2019 ▸)
*T*_min_, *T*_max_	0.860, 1.000
No. of measured, independent and observed [*I* > 3σ(*I*)] reflections	18695, 2211, 1432
*R*_int_	0.087
(sin θ/λ)_max_ (Å^−1^)	0.744

Refinement	
*R*[*F* ^2^ > 2σ(*F* ^2^)], *wR*(*F* ^2^), *S*	0.032, 0.037, 1.29
No. of reflections	2211
No. of parameters	124
Δρ_max_, Δρ_min_ (e Å^−3^)	0.61, −0.67

Computer programs: *CrysAlis PRO* (Rigaku OD, 2019 ▸), *SUPERFLIP* (Oszlányi & Sütő, 2004 ▸; Palatinus & Chapuis, 2007 ▸), *JANA2006* (Petříček *et al.*, 2014 ▸), *ATOMS* (Dowty, 2005 ▸).

## supplementary crystallographic information

### Crystal data

**Table:**

Na_6_Mg(SO_4_)_4_	*F*(000) = 540
*M**_r_* = 546.5	*D*_x_ = 2.682 Mg m^−^^3^
Monoclinic, *P*2_1_/*c*	Mo *K*α radiation, λ = 0.71073 Å
Hall symbol: -P 2ycb	Cell parameters from 3897 reflections
*a* = 9.7761 (6) Å	θ = 2.3–31.7°
*b* = 9.1998 (4) Å	µ = 1.04 mm^−^^1^
*c* = 8.2040 (5) Å	*T* = 296 K
β = 113.518 (7)°	Plate, colourless
*V* = 676.56 (7) Å^3^	0.03 × 0.02 × 0.004 mm
*Z* = 2	

### Data collection

**Table:**

SuperNova Rigaku Oxford Diffraction diffractometer with Pilatus200K detector	2211 independent reflections
Radiation source: micro-focus sealed X-ray tube, SuperNova (Mo) X-ray Source	1432 reflections with *I* > 3σ(*I*)
Mirror monochromator	*R*_int_ = 0.087
Detector resolution: 5.8140 pixels mm^-1^	θ_max_ = 31.9°, θ_min_ = 2.3°
ω scans	*h* = −14→14
Absorption correction: multi-scan (CrysAlisPro; Rigaku OD, 2019)	*k* = −13→13
*T*_min_ = 0.860, *T*_max_ = 1.000	*l* = −12→11
18695 measured reflections	

### Refinement

**Table:**

Refinement on *F*	0 restraints
*R*[*F*^2^ > 2σ(*F*^2^)] = 0.032	0 constraints
*wR*(*F*^2^) = 0.037	Weighting scheme based on measured s.u.'s *w* = 1/(σ^2^(*F*) + 0.0001*F*^2^)
*S* = 1.29	(Δ/σ)_max_ = 0.047
2211 reflections	Δρ_max_ = 0.61 e Å^−^^3^
124 parameters	Δρ_min_ = −0.67 e Å^−^^3^

### Fractional atomic coordinates and isotropic or equivalent isotropic displacement parameters (Å^2^)

**Table:**

	*x*	*y*	*z*	*U*_iso_*/*U*_eq_	
S1	0.14002 (6)	0.30539 (6)	0.22425 (8)	0.01023 (19)	
S2	0.66143 (6)	0.35283 (6)	0.34685 (8)	0.01116 (19)	
Mg1	0	0	0	0.0097 (4)	
Na1	0.31120 (11)	0.01426 (11)	0.46772 (12)	0.0173 (4)	
Na2	0.44443 (12)	0.15141 (12)	0.09037 (14)	0.0262 (4)	
Na3	0.88822 (12)	0.13860 (11)	0.31627 (14)	0.0231 (4)	
O1	0.03029 (18)	0.18470 (18)	0.1553 (2)	0.0153 (6)	
O2	0.13531 (19)	0.39708 (19)	0.0752 (2)	0.0169 (6)	
O3	0.0954 (2)	0.39427 (19)	0.3441 (2)	0.0197 (7)	
O4	0.28894 (18)	0.24382 (19)	0.3187 (2)	0.0198 (7)	
O5	0.66765 (19)	0.19352 (18)	0.3593 (2)	0.0175 (6)	
O6	0.6464 (2)	0.4160 (2)	0.5023 (2)	0.0208 (7)	
O7	0.80007 (17)	0.40614 (19)	0.3324 (2)	0.0154 (6)	
O8	0.53693 (18)	0.39792 (19)	0.1825 (2)	0.0190 (6)	

### Atomic displacement parameters (Å^2^)

**Table:**

	*U*^11^	*U*^22^	*U*^33^	*U*^12^	*U*^13^	*U*^23^
S1	0.0103 (3)	0.0099 (3)	0.0103 (3)	−0.0010 (2)	0.0039 (2)	−0.0008 (2)
S2	0.0109 (3)	0.0099 (3)	0.0126 (3)	0.0001 (2)	0.0046 (2)	0.0006 (2)
Mg1	0.0096 (5)	0.0090 (6)	0.0101 (5)	0.0002 (4)	0.0034 (4)	−0.0002 (4)
Na1	0.0195 (5)	0.0154 (5)	0.0181 (5)	−0.0002 (4)	0.0088 (4)	0.0005 (4)
Na2	0.0266 (6)	0.0187 (6)	0.0259 (6)	−0.0064 (4)	0.0026 (5)	−0.0002 (5)
Na3	0.0257 (6)	0.0206 (6)	0.0305 (6)	0.0040 (4)	0.0190 (5)	0.0039 (5)
O1	0.0156 (8)	0.0149 (9)	0.0167 (9)	−0.0076 (7)	0.0077 (7)	−0.0072 (7)
O2	0.0222 (9)	0.0155 (9)	0.0159 (9)	0.0009 (7)	0.0105 (7)	0.0041 (7)
O3	0.0267 (10)	0.0184 (10)	0.0189 (10)	−0.0037 (8)	0.0145 (8)	−0.0073 (8)
O4	0.0118 (8)	0.0182 (10)	0.0251 (10)	0.0011 (7)	0.0028 (7)	0.0031 (8)
O5	0.0223 (9)	0.0111 (9)	0.0195 (9)	0.0010 (7)	0.0088 (7)	0.0027 (7)
O6	0.0270 (10)	0.0198 (10)	0.0219 (10)	−0.0028 (8)	0.0165 (8)	−0.0052 (8)
O7	0.0095 (8)	0.0208 (9)	0.0144 (9)	−0.0026 (7)	0.0031 (7)	0.0005 (7)
O8	0.0113 (8)	0.0231 (10)	0.0174 (9)	0.0007 (7)	0.0003 (7)	0.0061 (8)

### Geometric parameters (Å, º)

**Table:**

S1—O1	1.4897 (17)	Na1—O8^i^	2.516 (2)
S1—O2	1.4710 (19)	Na1—O8^vii^	2.3504 (17)
S1—O3	1.472 (2)	Na1—O4	2.406 (2)
S1—O4	1.4630 (16)	Na3—O7	2.628 (2)
S2—O7	1.490 (2)	Na3—O1^viii^	2.305 (2)
S2—O5	1.4687 (18)	Na3—O5	2.374 (2)
S2—O8	1.4703 (15)	Na3—O2^i^	2.438 (2)
S2—O6	1.461 (2)	Na3—O2^ix^	2.5219 (18)
Mg1—O7^i^	2.0765 (15)	Na3—O3^i^	2.641 (2)
Mg1—O7^ii^	2.0765 (15)	Na3—O6^v^	2.7595 (18)
Mg1—O1	2.0732 (17)	Na2—O5	2.4368 (18)
Mg1—O1^iii^	2.0732 (17)	Na2—O8	2.447 (2)
Mg1—O3^iv^	2.099 (2)	Na2—O8^i^	2.945 (2)
Mg1—O3^v^	2.099 (2)	Na2—O4	2.969 (3)
Na1—O7^i^	2.4691 (19)	Na2—O4^v^	2.3460 (19)
Na1—O5^vi^	2.340 (2)	Na2—O6^i^	2.349 (2)
Na1—O2^vii^	2.369 (2)	Na2—O6^v^	2.438 (3)
			
O1—S1—O2	109.45 (9)	O7—Na3—O1^viii^	98.11 (7)
O1—S1—O3	107.87 (11)	O7—Na3—O5	57.17 (6)
O1—S1—O4	109.04 (10)	O7—Na3—O2^i^	138.97 (9)
O2—S1—O3	108.49 (11)	O7—Na3—O2^ix^	107.61 (6)
O2—S1—O4	110.90 (12)	O7—Na3—O3^i^	155.25 (6)
O3—S1—O4	111.03 (10)	O7—Na3—O6^v^	92.37 (6)
O7—S2—O5	108.68 (11)	O1^viii^—Na3—O5	146.76 (7)
O7—S2—O8	106.29 (11)	O1^viii^—Na3—O2^i^	122.88 (8)
O7—S2—O6	110.50 (11)	O1^viii^—Na3—O2^ix^	85.01 (7)
O5—S2—O8	109.97 (9)	O1^viii^—Na3—O3^i^	72.61 (7)
O5—S2—O6	110.59 (11)	O1^viii^—Na3—O6^v^	89.22 (7)
O8—S2—O6	110.70 (11)	O5—Na3—O2^i^	85.18 (7)
O7^i^—Mg1—O7^ii^	180	O5—Na3—O2^ix^	121.59 (8)
O7^i^—Mg1—O1	93.89 (6)	O5—Na3—O3^i^	119.63 (7)
O7^i^—Mg1—O1^iii^	86.11 (6)	O5—Na3—O6^v^	71.75 (7)
O7^i^—Mg1—O3^iv^	86.17 (7)	O2^i^—Na3—O2^ix^	76.82 (6)
O7^i^—Mg1—O3^v^	93.83 (7)	O2^i^—Na3—O3^i^	55.92 (7)
O7^ii^—Mg1—O1	86.11 (6)	O2^i^—Na3—O6^v^	90.26 (6)
O7^ii^—Mg1—O1^iii^	93.89 (6)	O2^ix^—Na3—O3^i^	94.63 (6)
O7^ii^—Mg1—O3^iv^	93.83 (7)	O2^ix^—Na3—O6^v^	159.78 (7)
O7^ii^—Mg1—O3^v^	86.17 (7)	O3^i^—Na3—O6^v^	65.16 (6)
O1—Mg1—O1^iii^	180	O5—Na2—O8	59.06 (6)
O1—Mg1—O3^iv^	89.68 (7)	O5—Na2—O8^i^	75.29 (6)
O1—Mg1—O3^v^	90.32 (7)	O5—Na2—O4	83.23 (7)
O1^iii^—Mg1—O3^iv^	90.32 (7)	O5—Na2—O4^v^	143.28 (8)
O1^iii^—Mg1—O3^v^	89.68 (7)	O5—Na2—O6^i^	121.73 (7)
O3^iv^—Mg1—O3^v^	180	O5—Na2—O6^v^	76.71 (7)
O7^i^—Na1—O5^vi^	99.95 (7)	O8—Na2—O8^i^	128.07 (7)
O7^i^—Na1—O2^vii^	114.43 (7)	O8—Na2—O4	76.04 (7)
O7^i^—Na1—O8^i^	56.73 (6)	O8—Na2—O4^v^	86.11 (6)
O7^i^—Na1—O8^vii^	142.59 (9)	O8—Na2—O6^i^	179.16 (7)
O7^i^—Na1—O4	86.12 (7)	O8—Na2—O6^v^	94.82 (8)
O5^vi^—Na1—O2^vii^	87.54 (8)	O8^i^—Na2—O4	75.26 (6)
O5^vi^—Na1—O8^i^	91.31 (7)	O8^i^—Na2—O4^v^	141.22 (7)
O5^vi^—Na1—O8^vii^	89.82 (6)	O8^i^—Na2—O6^i^	52.75 (6)
O5^vi^—Na1—O4	173.33 (8)	O8^i^—Na2—O6^v^	97.65 (7)
O2^vii^—Na1—O8^i^	170.73 (6)	O4—Na2—O4^v^	100.43 (7)
O2^vii^—Na1—O8^vii^	101.91 (7)	O4—Na2—O6^i^	104.22 (8)
O2^vii^—Na1—O4	87.42 (7)	O4—Na2—O6^v^	159.86 (6)
O8^i^—Na1—O8^vii^	87.27 (7)	O4^v^—Na2—O6^i^	93.06 (7)
O8^i^—Na1—O4	94.35 (8)	O4^v^—Na2—O6^v^	96.74 (8)
O8^vii^—Na1—O4	86.94 (6)	O6^i^—Na2—O6^v^	85.18 (8)

Symmetry codes: (i) −*x*+1, *y*−1/2, −*z*+1/2; (ii) *x*−1, −*y*+1/2, *z*−1/2; (iii) −*x*, −*y*, −*z*; (iv) −*x*, *y*−1/2, −*z*+1/2; (v) *x*, −*y*+1/2, *z*−1/2; (vi) −*x*+1, −*y*, −*z*+1; (vii) *x*, −*y*+1/2, *z*+1/2; (viii) *x*+1, *y*, *z*; (ix) *x*+1, −*y*+1/2, *z*+1/2.
